# Short-Term Economic Impact of COVID-19 on Spanish Small Ruminant Flocks

**DOI:** 10.3390/ani10081357

**Published:** 2020-08-05

**Authors:** Irene Vidaurreta, Christian de la Fe, Juan Orengo, Ángel Gómez-Martín, Bernardino Benito

**Affiliations:** 1 Department of Accounting and Finance, Faculty of Economics and Business, Regional, Campus of International Excellence “Campus Mare Nostrum”, University of Murcia, 30100 Murcia, Spain; irene.vidaurreta@um.es (I.V.); benitobl@um.es (B.B.); 2 Ruminant Health Research Group, Department of Animal Health, Faculty of Veterinary Sciences, Regional, Campus of International Excellence “Campus Mare Nostrum”, University of Murcia, 30100 Murcia, Spain; angelgomez@um.es; 3 Department of Animal Production, Faculty of Veterinary Sciences, Regional, Campus of International Excellence “Campus Mare Nostrum”, University of Murcia, 30100 Murcia, Spain; jorengo@um.es; 4 Microbiological Agents Associated with Animal Reproduction (ProVaginBio) Research Group, Department of Animal Health and Public Health, Faculty of Veterinary Sciences, University CEU Cardenal Herrera of Valencia, CEU Universities, 46113 Valencia, Spain

**Keywords:** COVID-19, small ruminants, economic impact, dairy, meat

## Abstract

**Simple Summary:**

The human pandemic COVID-19 has rapidly spread around the world, leading to extreme control measures such as population confinement and industries activity closure, including tourism and restauration. Although small ruminants are not sanitary affected, this situation might cause a negative economic impact on Spanish flocks. The data analyses provided by producers and slaughterhouses in the initial 60 days after COVID-19’s pandemic declaration, showed that while the dairy goat flocks suffered a substantial drop in milk prices, this impact was not seen in sheep milk, which remained almost stable. A price drop for lambs or goat kids was also reported. These data are in agreement with the unexpected drop of lambs and goat kids’ sacrifices in April, as reported by some slaughterhouses. We registered a short-term negative economic impact on Spanish small ruminant’s flocks after COVID-19’s pandemic declaration in the country. The long-term economic consequences still need to be studied to establish contingency plans for this type of sanitary crisis.

**Abstract:**

The human pandemic COVID-19 caused by the severe acute respiratory syndrome coronavirus-2 (SARS-CoV-2) started in China in 2019 and has rapidly spread around the world, leading to extreme control measures such as population confinement and industry activity closure. Although small ruminants are not sanitary affected by this virus, the short-term economic impact derived by COVID-19 on Spanish flocks is estimated in this study, using data provided by producers and two major slaughterhouses. Milk prices of dairy goat flocks suffered a substantial drop in April 2020, close to 4.5 cts EUR/liter compared to the previous month. In contrast, the monthly milk prices in sheep remained almost stable during this period, and even increases of more than EUR 6 cts were reported in comparison with the previous year. Nevertheless, economical differences are reported by areas where producers could receive a higher income, close to EUR 0.3 per liter of milk. Global data provided by feedlots affecting 2750 Spanish flocks evidenced a lamb price drop ranging from 16.8% to 26.9% after the pandemic arrival; in line with the data directly reported by a limited sample of producers (ranging from 11.0% to 23.7%). The goat kid meat market also suffered a reduction in prices per kg, near 12.5%; although, for some flocks, losses reached up to 40%. In the same line, 2 slaughterhouses reported a sudden sacrifice drop around 27% for lambs and goat kids sacrifices in April, in contrast with the usual sacrifice figures from the beginning of 2020. Moreover, our study showed a temporary and unexpected retention of lambs and goat kids at farms due to a reduction in animals slaughtered during this period. In conclusion, data evidenced a considerable negative economic impact on Spanish small ruminant flocks, throughout the first 60 days after COVID-19’s pandemic declaration. Further studies are needed to evaluate the long-term economic consequences, in order to establish contingency plans and avoid the collapse of small ruminant industries when a crisis of these characteristics occurs.

## 1. Introduction

A new pandemic, COVID-19, caused by the severe acute respiratory syndrome coronavirus-2 (SARS-CoV-2) started in China in December 2019 and has rapidly spread around the world [1]. As of June 22, 2020, more than 9 million cases of COVID-19 occurring in at least 188 countries and territories were reported [2]. This is the third zoonotic coronavirus that has emerged in the last two decades after the severe acute respiratory syndrome CoV (SARS-CoV) occurred in Asia between 2002 and 2003, and the Middle East respiratory syndrome coronavirus (MERS-CoV), first described in Saudi Arabia in 2012 [3,4].

COVID-19 was officially declared in Spain in February 27, 2020, and near the end of June, more than 247,000 diagnosed human infections and more than 28,000 deaths were confirmed [5]. SARS-CoV-2 infection required a high number of intensive care places for treatment of the most serious pneumonia cases. Extreme control measures such as population confinement, social activities interruption or industries’ temporary closures were adopted by Spanish authorities on March 14, aiming at halting the number of new infections [6]. Sheep and goat farms were considered economically essential activities. It involved a social and economic impact on most sectors, which is difficult to estimate.

Spain is one of the most important small ruminant producers in Europe [7], being considered the third in total population of sheep and goats behind Greece and France, respectively. Small ruminant industries are based on milk and/or meat production with local differences linked to heritage, touristic and gastronomic particularities. Cheese production is very high in Spain [8], and almost all small ruminants’ milk production goes directly to the cheese industry [9,10]. Although lamb and goat kid production is the main income for meat producers, it also represents a secondary input for dairy small ruminant farmers.

No virus or minimal shedding was detected after the intranasal inoculation of young goats and adult sheep with MERS-CoV [11], thus suggesting that ruminants are not susceptible to the new virus SARS-CoV-2. Although COVID-19 cannot be considered a sanitary problem for these species, the pandemic impact should be analyzed. In fact, the population confinement and the sudden disruption of gastronomic activity have led to a new global context [5]. This situation could affect the demand and consumption of small ruminant products, having a relevant economic impact on flocks, either short or long-term. According to our hypothesis, some surveys showed that 49.2% of the population in Spain experienced “a lot” or “quite a lot” of negative lifestyle impacts on their household. Specifically, 8%, 12% and 22% had “a lot”, “quite a lot” and “somewhat” negative financial impact on their economies [12].

A short-term economic impact of COVID-19 on Spanish small ruminant flocks is estimated based on data provided by producers of the most important areas of the country in the first 60 days after COVID-19’s pandemic declaration (February 27, 2020). The information related to milk and meat production from January to April 2020 (and the same period of the previous year), as well as the revenues, were reported by farmers using technical–economic management softwares. Pairwise comparisons between months and years (intra- and interannual) were performed, and then differences were discussed.

## 2. Materials and Methods

This is an initial assessment of the short-term economic impact based on the first two months after the imposed population confinement. This study was based on the information provided by farmers and two of the major small ruminants’ slaughterhouses:

### 2.1. Farmers

**Figure 1:**
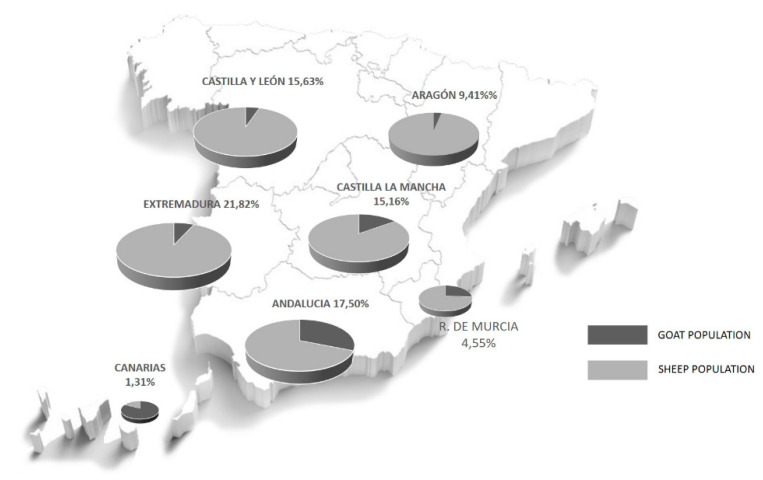
Sheep and goat populations in the studied areas, involving over 80% of the total population [13].

Productive and economic data were taken from a total of 60 Spanish farms (*n* = 35 sheep farms and *n* = 25 goat farms). Farms were sampled from among those that use some technical–economic management software, and whose farmers and technicians volunteered their data for this study (*n* = 14, 14, 14, 9, 7 and 2 farms from Castilla-Leon, Castilla-La Mancha, Andalusia, Murcia, the Canary Islands and Catalonia, respectively). Sheep and goat populations in Spain were estimated in 15–16 and 3 million heads, respectively [9]. Central regions of the country (Castilla-La Mancha and Castilla-Leon) comprise more than 80% of Spanish dairy sheep, and thus, they represent most of the flocks included in this study (Figure 1). Data from meat sheep flocks of Castellana and Segureña breeds were collected to evaluate the impact on this subsector. On the other hand, Andalusia (40%), the Canary Islands, Murcia and Castilla-La Mancha represent more than 70% of dairy goat flocks, as well as the most abundant breeds of the country (Murciano-Granadina, Malagueña or Majorera) [9], covering most of the flocks sampled in this study. Data were the average monthly price per liter of milk, and prices per kilogram of lamb or goat kid produced and sold, from January to April 2019 and 2020.

On the other hand, lamb meat production was also complemented with the information provided by the feedlots of Extremadura, Aragón and other areas, which clusters lamb production of many breeds and sizes. In fact, meat sheep products are different based on weight at slaughter and final destination; thus, producers send lambs to specialized feedlots in which the animals reach the adequate commercial size. For confidentially reasons, feedlots provided the variation rate of prices for the Post-COVID period regarding Pre-COVID months, instead of the monthly prices provided by producers. Consequently, the price variation during the research period in 2020 and 2019 was also compared, according to the type of product and geographical area. These data are representative of the producers’ income clustering approximately 2750 flocks in Spain (about 3% of the flocks in the country) [9].

### 2.2. Slaughterhouses

The source of this information was the number of carcasses processed per month from two major slaughterhouses with capacity to sacrifice more than 50,000 animals per year, commercially focused on the national market, and both placed in South East Spain. Their activities during these months of 2019 and 2020 (%) were then compared.

### 2.3. Statistical Analysis

The economic situation of small ruminants’ sector was analyzed using the information reported by different flocks monitored with technical-economic management softwares. The factors of the study were: livestock species (either sheep or goat), breed and geographical area by species and productive purposes and system (milk and/or meat). The variables were the unit prices for milk and meat paid to the producer (EUR/L and EUR/kg, respectively). The monthly prices of January–April for the last 2 years (2019–2020) were analyzed by SPSS.24 (IBM SPSS Statistics, New York, USA), and used to perform pairwise comparisons between months and years (intra- and interannual). Therefore, two tailed paired t-tests were used to prove either a “change” or a “difference” in monthly prices across time (“within-flocks” observations). The significance level was set at *p* < 0.05.

## 3. Results

### 3.1. Analysis of Milk Prices

**Figure 2:**
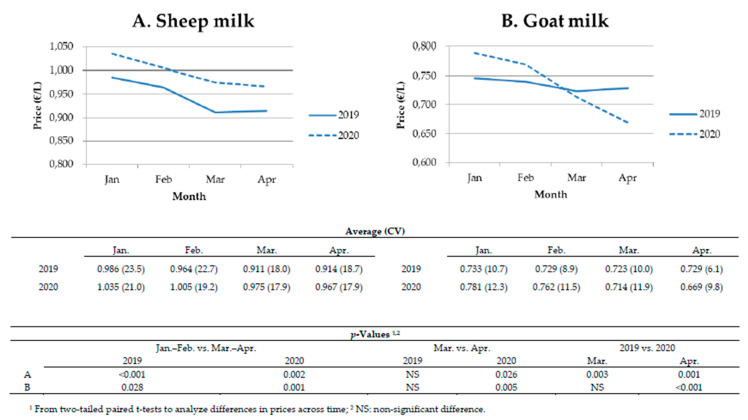
Average monthly price per liter (EUR/L) of sheep (**A**) and goat milk (**B**) reported by producers from January to April 2019 and 2020 (coefficient of variation, %), with intra- and interannual comparisons. Data from 23 and 22 dairy sheep and goat flocks, respectively.

Considering the market of milk in 2019 and 2020, the analyses of prices (EUR/L) showed an expected seasonal drop in March–April compared to January–February in both species (Figure 2). Moreover, significant differences were shown for the intra-annual prices comparing March vs. April for 2020. These differences for sheep were not relevant in relative terms (1%). However, the drop of prices reached approximately EUR 4.5 cts per liter for goat flocks, representing a decrease of more than 6% in comparison with the previous month. For the interannual prices, differences were significant in April for both species. The results showed an increase in the revenues received by dairy sheep flocks, but the prices for goats decreased (Figure 2). Indeed, the producers of sheep milk received EUR 6.4 and 5.3 cts per liter more in March and April 2020, respectively, in comparison with the same months of 2019; conversely, the interannual data for April showed a decrease in goat milk price (EUR 6 cts per liter) representing over 8%.

**Figure 3:**
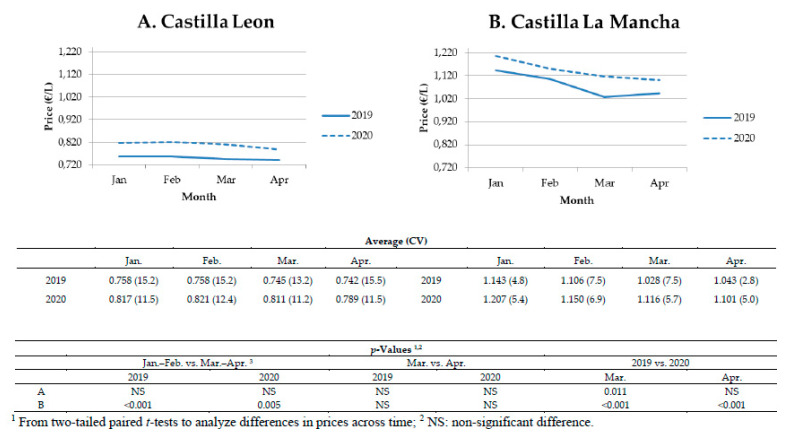
Average monthly price per liter of sheep milk (EUR/L) reported by producers from January to April 2019 and 2020 according to area (coefficient of variation, %), with intra- and interannual comparisons. Data from 21 dairy sheep flocks.

**Figure 4:**
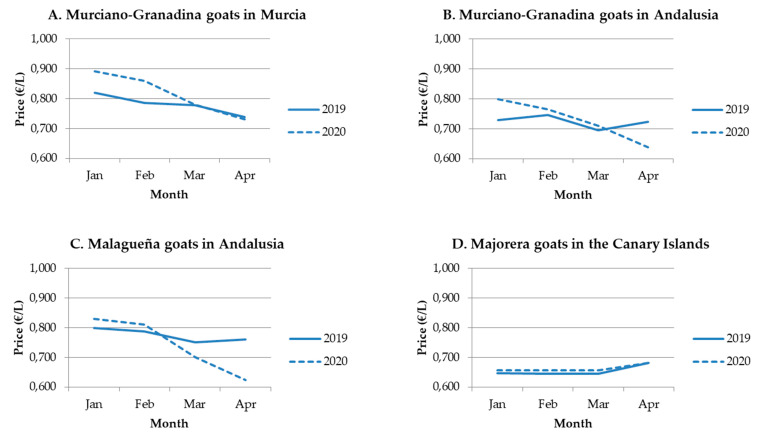

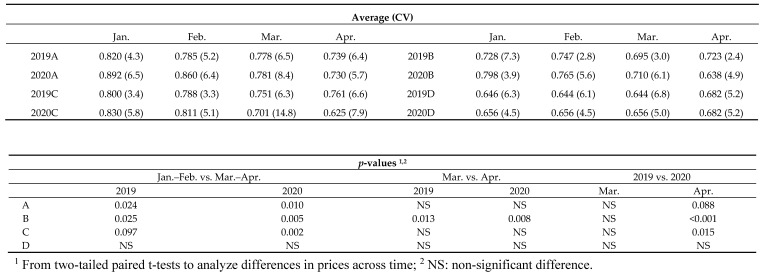
Average monthly price per liter of goat milk (EUR/L) reported by producers from January to April 2019 and 2020 (coefficient of variation, %) according to area and breed, with intra- and interannual comparisons. Data from 20 dairy goat flocks.

When the prices of sheep milk were analyzed, considering the two main geographical locations for dairy sheep in Spain (Figure 1), results showed differences among flocks (Figure 3). The general drop in milk prices (March–April vs. January–February) was only found in Castilla La Mancha in both years. Regarding March and April, in general, interannual, but not intra-annual differences were found, regardless of where the flock was located. Although results confirmed the increase in prices for sheep milk (Figure 2), this interannual effect was non-significant in Castilla Leon in April (Figure 3). Moreover, the flocks established in Castilla-La Mancha vs. Castilla Leon received a higher revenue (close to 0.3EUR/liter), in whatever month or year studied. In this sense, the coefficient of variation of monthly prices in Castilla-La Mancha ranged from 2.8% to 7.5%, confirming that the prices are similar in all the flocks as per this study. For goat milk, as we previously described for March–April in comparison with January–February, most of dairy goat flocks studied showed either a negative tendency or a reduction in milk prices in both years, except for the Canary Islands (Figure 4). Despite recording numerically higher monthly prices in early 2020 vs. 2019, the interannual comparison for April showed a price drop in most of the areas and breeds studied (Figure 4). These reductions ranged from 9 to 14 cts EUR/liter for goat flocks placed in Andalusia. Moreover, a substantial drop in prices was registered in 2020 when April vs. March was compared (intra-annual comparison). Although there were apparently relevant price differences, a significant effect was only recorded for the milk prices of Murciano-Granadina flocks placed in Andalusia; with differences near EUR 7 cts per liter (−7.1 ± 0.02 cts EUR/L).

### 3.2. Analysis of Meat Prices

The price analyses of lamb (EUR/kg) in 2019 and 2020 showed a higher unexpected price drop in March–April 2020 (Table 1). While the regular prices usually drop in some areas of the country during this period, this decrease was more pronounced this year. In fact, the variation rate (%) of prices between the pre- and post-COVID periods was higher in 2020 compared to 2019; these percentages were always negative in 2020 (Table 2). The price drops registered for lambs using the data provided by producers (Table 1) ranged from 10.8% to 23.7% (from −0.33 to −0.87 EUR/kg, respectively), while these differences ranged from 16.0% to 26.9% using information involving 2750 flocks (Table 2).

Goat kid meat also suffered a decrease in prices. Producers sell goat kids with similar sizes, so prices are comparable among geographical areas. Statistical analyses showed a price mean decrease close to 12.5% (−0.49 ± 0.177 EUR/kg; *P* = 0.017), although losses reached up to 40% of the regular price in some flocks (data not shown).

### 3.3. Analysis of Slaughterhouses Data

The interannual comparison of sacrifices (2020 vs. 2019) in 2 slaughterhouses showed a drop in activity in April (−28.5% and −25.9% for goat kids and lambs, respectively; Table 3). In contrast, a higher number of animals sacrificed was reported by the 2 slaughterhouses in March 2020 in comparison with 2019—in agreement with the positive growth percentages of January and February. For the intra-annual comparisons (April vs. March), a drop of 39.0% and 41.3% in the number of lambs and goat kids sacrificed was observed in 2020; whereas in 2019, the decrease for the same period was of 15.8% and 2.9%, respectively.

## 4. Discussion

A sudden and direct economic impact associated with the COVID-19 pandemic in Spanish small ruminant flocks is observed between March and April 2020. Direct consequences for the revenues on dairy and meat markets are evident from a general point of view. Nevertheless, differences were also remarked depending on the ruminant species and the geographical area. The influence of other factors such as the participation in cooperatives or the presence of Protected Designation of Origin (PDO), which was out of scope in this study, should be further analyzed.

The low consumption of bulk milk and milk derivatives from goat and sheep milk, compared to cow milk [14], leads us to consider the vulnerability of this dairy sector to the restrictions of COVID-19, but the short-term effects were mainly noticed in dairy goats. This way, the prices of milk for dairy sheep flocks (represented by the most important breeds such as Spanish Assaf (Assaf.E), Churra or Manchega [15,16]), remained almost stable during the immediate post- COVID-19 period in 2020. This effect is probably due to the contractual relationship of many flocks with big enterprises responsible for milk and cheese production and distribution. The presence of international quality products such as “Manchego cheese”, associated with Protected Designation of Origin (PDO) flocks [17], surely helped to maintain high prices. However, the effect of the pandemic was more pronounced in some flocks, in which the reduction in prices, or even the sudden contract cancellation, forced farmers to “trash” the milk and dry animals. Our data suggest that the Spanish sheep flocks linked to a PDO, which implies that particular characteristics are present in the product [18], could be less vulnerable to the negative effects caused by this pandemic.

From a general point of view, dairy goat flocks were probably hit the most by revenue reductions. In spite of this, differences between areas and flocks were evident, therefore suggesting the absence of common strategies by producers and highlighting non-collective price negotiation. However, the prices of goat milk remained stable in the Canary Islands, where the cheese consumption per capita is the highest in the country [19], which could explain this trend. In our view, factors that could explain the specific negative effect on goat dairy flocks would be: 1- Goat milk is frequently used to prepare yogurts and ice creams [14], frequently used to make desserts in restaurants that have been closed during the first phase of the pandemic in Spain. 2- It presents little diversity in the type of consumer, since there are consumers who reject the flavor associated with goat products [20].

Considering the evolution of small ruminant milk prices, a seasonal effect can be noted where regular prices usually drop from January to April. However, this decrease was more pronounced in comparison with the previous year in goat milk, taking into account that the highest goat milk price in recent years was recorded in early 2020 according to InLac (Spanish Interprofessional Dairy Organisation). On the other hand, although the evolution of prices after the Spanish deconfinement plan (approved on April 28 [21]) is not still fully known, preliminary data for May and June obtained from some farms point out a stabilization of the market price of milk. However, the aim of our research was not, as stated above, to study the evolution of the milk market, but the COVID 19 effect for the initial confinement period.

COVID-19 directly affected the meat market, although its impact was not equal across all flocks as there is a dependency on several factors. In fact, meat sheep products are different based on the weight at slaughter and their final destination (national or international markets). Moreover, other commercial factors linked to the presence of quality marks also can significantly influence occasional consumers [22]. Nevertheless, it is necessary to remark that: 1) COVID-19 was declared in Spain when the lamb prices were cheaper than other seasons, but the pandemic increased the price fluctuation. In this sense, this seasonality factor in the small ruminant production and prices should be considered for longer research periods; 2) On the other hand, it is remarkable that the feedlots and cooperatives continued to collect animals from the flocks, avoiding the commercial collapse. Additionally, it is very interesting to analyze what happened with the Segureña breed, linked to the traditional consumption of high-quality lamb [23]. They started an aggressive publicity action focused on the consumption of this appreciated lamb at cheaper prices; including cost free home delivery [24]. Moreover, such promotions could be of benefit if we consider that baking is a very popular eating habit in Spain [25]. As a result, sales remained stable. However, the number of lambs sacrificed dropped in April 2020 (Table 3, confirming that COVID-19 has damaged a promising year for lamb production.

The disadvantage of goat kid over lamb production is that the caprine products should be consumed after 1 month of life, while lambs could be fattened during a longer time period, therefore allowing a longer commercial life. Nonetheless, distribution channels were suddenly disrupted because of the demand drop, thus reflecting the price decrease; along with a reduction in animals sacrificed over 30%.

Overall, sheep and goat food products should be considered highly vulnerable to events likeCOVID-19. Given the generalized “dinning-out” tradition in Spain, consumers prioritize social meals over individual ones [26]. These habits changed during the first months of COVID-19 when an increase in snacks and ultra-processed food consumption was found [27]. Sheep and goat products are not among the long-storage foods that the population compulsively bought at the beginning of pandemic (long-life milk, pasta, rice and tinned vegetables) [27].

It was recently suggested that animal health could be affected by immediate human confinement measures and by the long-term economic consequences of COVID-19 [28]. In this sense, our study showed a temporary and unplanned retention of lambs and kids at some farms due to a reduction in animals slaughtered. Unsold lambs and goat kids not collected on time led to increased animal density and microbiological load at farms. This can also be the reason for adverse health effects on these collectives, due to a higher prevalence of respiratory diseases such as mycoplasmosis [29,30]. This could also be aggravated due to the halt of some national eradication programs (such as contagious agalactia [31]) against diseases, related to the COVID-19 non-essential activities stop [26]).

## 5. Conclusions

Data provided by producers showed the negative economic effects in the initial 60 days after COVID-19’s pandemic declaration. Considering that data were provided by farms using technical–economic management software, it could be possible to consider a worse scenario for non-monitored flocks. Further studies are needed to evaluate medium and long-term economic consequences of this event, where other factors such as the quality of PDO products and the seasonality of small ruminant production and prices, are taken into account. Small ruminant industries should consider market globalization and potential recurrence of new waves of this pandemic to elaborate contingency plans in order to avoid the economic collapse of this sector. The e-commerce boom triggered by post-COVID-19, as well as the search of new distribution channels and market opportunities, should also be useful options to be explored. Policymakers and governments should consider the effects that new confinements could have on this essential sector, in order to articulate the needed economical aids.

## Figures and Tables

**Table 1:** Average monthly price per kilogram of lamb (EUR/kg) reported by producers for March–April 2019 and 2020 (coefficient of variation, %) according to breed and location, with interannual comparisons. Data from 31 sheep flocks.

Breed (Location)	Mar.-Apr. 2019	Mar.-Apr. 2020	*p*-Values ^1^
			2019 vs. 2020
Assaf, Churra and Castellana (northeast)	3.67 (10.0)	2.80 (10.8)	<0.001
Manchega (central)	4.07 (14.3)	3.22 (18.8)	<0.001
Segureña (southeast)	3.11 (0.0)^2^	2.78 (0.0)^2^	-

^1^ From two-tailed paired t-tests to analyze differences in prices across time. ^2^ The same monthly prices for all producers (grouped by Protected Designation of Origin, (PDO).

**Table 2:** Variation rate (%) of prices paid to producers in 2019 and 2020 per area (covering main breeds) and type of lamb (by weight at slaughter), as well as the number estimated of flocks involved.

Area (Location)	Main Breeds	Lamb Weight (kg)	Number of Flocks (*n*)	2019(%) ^1^	2020(%) ^1^	2020 vs. 2019(%)
Extremadura (southwestern)	Merino	23	1800	2.7	−19.0	−21.7
Valle del Ebro (northeast)	Rasa aragonesa, Navarra, Ojalada, INRA401 and others	25		1.0	−28.0	−29.0
Valle del Ebro (northeast) ^2^	Rasa aragonesa, Roya bilbilitana, Ojalada de Teruel	22		0.2	−17.0	−17.2
Castilla Leon(central)	Different breeds or crosses	16	850	−5.1	−32.0	−26.9
Aragon-Navarra (northeast)	Rasa aragonesa, Roya bilbilitana, Ojalada de Teruel, Churra tensina o xisqueta, Rasa navarra	12		−5.0	−21.0	−16.0
Castilla La Mancha(central)	Manchega	12		−2.0	−24.0	−22.0
Andalusia-Murcia (southeast)	Segureña	20	100	3.0	−17.5	−20.5

^1^ Price variation by year was calculated as follows: (1) $\left(\frac{Price^{Mar14/Apr30}}{Price^{Jan1/Mar13}}-1\right)\times 100$
where $Price^{Mar14/Apr30}$ was the average price per kilogram from March 14 to April 30 (post-COVID period), and $Price^{Jan1/Mar13}$ was the average price per kilogram from January 1 to March 13 (pre-COVID period). ^2^ Lambs marketed under Protected Designation of Origin (PDO).

**Table 3:** Number of animals sacrificed by month in 2019 and 2020 from 2 small ruminant slaughterhouses, with intermonthly (April vs. March) and interannual (2020 vs. 2019) comparisons calculated as a percentage of change from baseline (%).

	2019					2020					2020 vs. 2019 (%)			
	Jan.	Feb.	Mar.	Apr.	Apr. vs. Mar. (%)	Jan.	Feb.	Mar.	Apr.	Apr. vs. Mar. (%)	Jan.	Feb.	Mar.	Apr.
Goat kids ^1^	4578	4879	5832	4912	−15.8	5022	5124	5980	3510	−41.3	9.7	5.0	2.5	−28.5
Lambs ^1^	1027	1379	3886	3775	−2.9	1744	2076	4582	2797	−39.0	69.8	50.5	17.9	−25.9

^1^ Data from two major slaughterhouses placed in SE Spain, commercially focused on the Spanish market.
